# Hydraulic expansion facilitates remodeling of arteriovenous fistulas without increasing venous intimal hyperplasia in rabbits

**DOI:** 10.2478/abm-2021-0028

**Published:** 2021-10-29

**Authors:** Wanjun Ren, Jiyuan Niu, Yuejuan Du, Huili Jiang

**Affiliations:** Department of Nephrology and Blood Purification Center, Jinan Central Hospital, Cheeloo College of Medicine, Shandong University, Jinan 250013, Shandong Province, China; Department of Nephrology, Linyi Central Hospital, Linyi 276400, Shandong Province, China

**Keywords:** arteriovenous fistula, hyperplasia, rabbits, transforming growth factor beta1, tunica intima

## Abstract

**Background:**

An arteriovenous fistula (AVF) is considered essential for chronic hemodialysis.

**Objective:**

To determine the effects of hydraulic expansion on the intimal hyperplasia of an AVF.

**Methods:**

We divided 12 healthy male New Zealand white rabbits into a control group (vein without special handling and direct anastomosis with an artery, n = 6) and a hydraulic expansion group (vein dilated by hydraulic pressure before anastomosis, n = 6). Histopathomorphology was examined with hematoxylin and eosin staining and immunohistochemistry. Analysis of covariance (ANCOVA) was used to compare the data between the groups.

**Results:**

Immediately and 1 day after surgery, the diameter of the fistula vein in rabbits in the hydraulic expansion group was significantly larger than it was in the control group (*P* = 0.02 and 0.03 respectively), but not on subsequent days. After hydraulic expansion and before construction of the fistula, the wall of vein was noticeably thinner on macroscopic observation, and the anterior and posterior walls were indistinguishable. At 3 weeks after surgery in the hydraulic expansion group, cells in the vein wall were disordered, there were fewer elastic fibers, tissues from the endothelium to tunica externa were less dense, and there was less extracellular matrix than in the control group. Expression of connective tissue growth factor in the hydraulic expansion group was significantly less than that in the control group (*P* = 0.01). No differences were found in intimal thickness or immunohistochemistry scores for transforming growth factor-β1 between the groups.

**Conclusion:**

Hydraulic expansion did not increase intimal hyperplasia of an AVF, but facilitates remodeling of AVFs in rabbits.

An arteriovenous fistula (AVF) is essential to provide optimal chronic hemodialysis [1]. If an AVF cannot ensure normal hemodialysis, it may threaten the life of a patient with uremia. An AVF is considered the criterion standard for hemodialysis access as it has better patency rates and fewer complications than other access types [2]. Native AVF is the first choice when creating vascular access among hemodialysis patients [3], and is the preferred modality for longer survival and functional patency. The primary patency rate of successful constructed native AVFs is as high as 80.2% [4]. Poor condition of a blood vessel may result in postoperative fistula occlusion, delay or inhibition of fistula maturation, or poor blood flow.

Dilation of a vessel with a balloon catheter is an effective and important diagnostic and therapeutic method for stenosis, and is usually performed under endoscopy or radiography [5]. In a retrospective study [5], endoscopic check-up was used before and after balloon catheter dilation to determine the location, extent and the mean diameter of the stenosis, and the changes after expansion. Despite of the risks of vessel dilacerations and hemorrhage, balloon catheter dilation is relatively safe and effective in vessel with small lumens such as AVF [6, 7]. However, balloon catheter dilation cannot be performed in some instances of severely rigid vascular sclerosis, which cannot be expanded easily.

A new technique for hydraulic expansion of a vein and artery using a tube from a disposable venoclysis needle for our model of AVF was inspired by two articles describing finite element analysis of minimum hydraulic expansion pressure in industrial applications [8, 9]. The authors assumed that hydraulic expansion could reduce the reflow resistance of proximal tubes and improve their patency [8, 9]. Hydraulic expansion was assumed in vascular sclerosis because the characteristics of the blood vessels and blood are similar to the tubes and fluids in industrial applications. To our knowledge, models of AVFs to study changes in the hemodynamic shear force in internal fistulas have been mainly created in large animals such as pigs and sheep, while small animals such as rabbits and rats have been used to study molecular and cyto-pathological mechanisms. Compared with rats, rabbits have larger blood vessels and are easier to use for surgery. Other models of AVF in rabbits have been reported elsewhere [10, 11]. The current model of AVF in rabbits was constructed to determine the effects of hydraulic expansion on the intimal hyperplasia of an AVF.

## Methods

### Materials

The present study was conducted according to the Regulations of the Administration of Laboratory Animals formulated by the People’s Republic of China National Science and Technology Commission, the Guiding Opinions on the Good Treatment of Laboratory Animals issued by the Ministry of Science and Technology, and National Laboratory Animal Management Regulations (1988). The animal experimental protocols were approved by the Medical Ethics Committee of Jinan Central Hospital (2016.3.1). We followed the ARRIVE 2.0 reporting guidelines [11].

A model of jugular autogenous AVF in rabbit was followed [12]. Healthy male New Zealand white rabbits (provided by the Jinan Xilingjiao breeding center, laboratory animals quality certificate No. 0001084) with weight of 2.0–2.5 kg (age 3–4 months) were chosen for the present study. The rabbits were fed with a freely available diet and housed in single cages at ambient room temperature and humidity of 40%–60%. Sample size was set to 6 using a resource equation method. We used 12 New Zealand white rabbits numbered from 1 to 12, and 12 continuous random numbers were obtained from a random digit table corresponding to the rabbit number. On the basis of random numbers, rabbits were divided into a control group (vein without special handling and anastomosis with the artery directly, n = 6) and a hydraulic expansion group (vein was dilated with hydraulic pressure at about 8.41 kPa before anastomosis with the artery, n = 6).

### Construction of AVF model in rabbits

**Figure 1:**
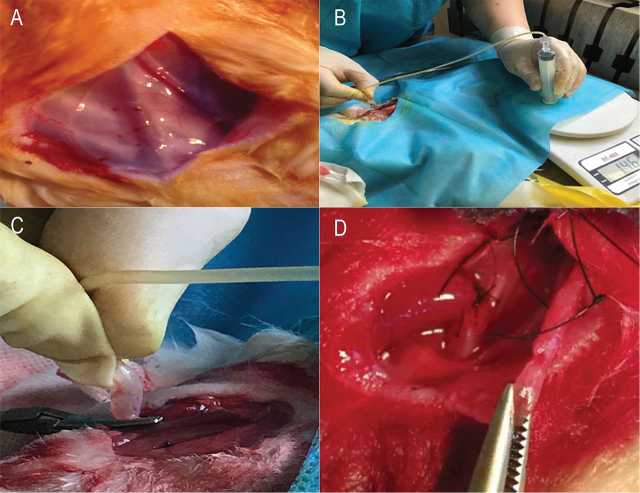
Surgical procedure. **(A)** Internal jugular vein, common carotid artery, and external jugular vein of rabbit. **(B)** Vein dilatated with hydraulic pressure. **(C)** Vein dilatated with hydraulic pressure (close-up). **(D)** Successfully constructed fistula.

New Zealand white rabbits were fed ad libitum for 3 days. Appetite and activities of rabbits were observed. A healthy rabbit having good appetite and behavioral activities was selected and then fasted for 8 h before surgery. An operator possessing relevant qualifications performed the procedures under license from the Shandong Experiment Animal Society of China. A dose of 1% sodium pentobarbital was used for anesthesia was based on the preoperative weight (30 mg/kg). The sodium pentobarbital was infused through an ear vein as the ear veins of rabbits are relatively superficial and easy to access. A sign of a suitable plane of anesthesia was that the rabbit’s limbs were weakened and did not respond to a pinch of the skin and respiration was deep. The limbs of the rabbit were fixed with the head supine and its jugular vein exposed by removing cervical fur. The internal jugular vein and carotid artery were scanned before and after surgery and the diameter of vein was recorded. The skin of neck was lifted slightly with forceps, and then the skin was incised with general tissue scissors along the right of the center of the neck. If the rabbit was observed to respond to the incision, 1% lidocaine was added additionally by local infiltration so that anesthesia was maintained for up to 2.5 h. The confluence of the internal and external jugular vein is the common jugular vein, so we could clearly identify the vein. We anastomosed the right common jugular vein and right common carotid artery. The arterial pulse between the vein and trachea was palpated, and deep layer muscles were bluntly dissected along the edge of the thyroid so as to find the internal carotid artery (**Figure 1A**). The artery was separated from the vein. The bleeding point and branch were ligated and the distal end of vein was ligated.

In rabbits of the hydraulic expansion group, the proximal part of vein was expanded by hydraulic pressure of about 8.41 kPa from heparin saline in the tube of a disposable venoclysis needle attached to a syringe, and the degree of expansion was such that the duct of vein was transparent (**Figure 1B** and **1C**) and maintained for 30 s. Heparin saline was injected after expansion. In the control group, heparinized saline was injected into the vein directly. The distal end of artery was ligated and heparinized saline was injected into the proximal artery. The diameter of vein was about 2 mm and the diameter of artery was about 1.6 mm. The venous and arterial proximal parts were occluded using a vessel clamp (hemostat). The clamp was placed 2 cm above the anastomotic stoma. The proximal artery and vein were anastomosed in the way of the end-to-end gap with 6-0 suture silk to ensure a good flexibility of the blood vessel after anastomosis, less angular deformity, and less impact on the laminar flow of blood. The “sling with four thread” method was used to suture at 12, 3, 6, and 9 o’clock points at first, then the four threads were tightened, and the artery and venous gap was anastomosed until the anastomosis was complete. The fistula was created successful with tremor and bruit after releasing the clamp (**Figure 1D**). It was thus evident that the anastomotic stoma was in a good apposition without hemorrhage or severe angular deformity. Blood filled the vein, without high tension and tremor could be felt by palpation. The vascular murmur bruit was continuous and soft during auscultation. If there was hemorrhage from the stoma, the anastomosis was reinforced with 7-0 or 10-0 suture silk. If there was no active bleeding, the skin was sutured. The diameter of the internal jugular vein was measured using ultrasound. The respiration of rabbit was observed and if not labored the rabbit was returned to its cage. The rabbits were put in a quiet environment after the surgery, and buprenorphine was used for analgesia, 0.05–0.1 mg/kg, subcutaneously. Based on the rabbit’s appetite and activity, the analgesia was administered every 8–12 h, for 1–2 days. B-mode ultrasonic diagnostic equipment (mSonics MU1L; with Probe L10-5; Chengdu Youtou Technology, Sichuan, China) was used to measure venous diameter of rabbit before surgery, during surgery, and 1 day, 2, 3, 7, 14, and 21 days after surgery.

### Specimen collection

The investigators who conducted specimen collection, hematoxylin and eosin (HE) staining, and the immunohistochemistry were blinded to the grouping. Venous samples of about 5 mm with and without hydraulic expansion were obtained during surgery. Samples were fixed in 4% paraformaldehyde for 12–24 h and stored at room temperature before use. At 21 days after surgery, rabbits were anesthetized with 1% pentobarbital sodium (30 mg/kg) again and 10–20 mL of air was directly injected into the internal iliac vein during anesthesia causing air embolism used for euthanasia [13, 14]. The vessels of the fistula were found along the previous cut, they were separated, and a 2 cm venous sample was obtained after the anastomotic stoma. Samples were fixed in 4% paraformalde-hyde for 12–24 h and stored for use.

### Hematoxylin and eosin (HE) staining

The venous tissue sample was rinsed with distilled water for 20 min and then dehydrated with alcohol for 30 min. The sample was embedded in molten wax at 58 °C with the venous lumina arranged verticality and a paraffin wax block was formed. The bulk paraffin was sliced off and the thickness of the slice was adjusted to 4 μm, with uniform thickness, integrity, and no defect in the tissue. Then the tissue sections were placed into 45 °C constant temperature waterbath, and attached to a microscope slide. A pencil was used for labeling. The slide was placed into an oven, baked at 65 °C for 30 min, and prepared for staining.

Tissue sections were routinely deparaffinated with xylene and alcohol. Hematoxylin staining using hematoxylineosin staining solution (catalog No. BSBA-4027; Zsgb-Bio, Beijing, China) was performed for 5 to 10 min, and the slide was rinsed with tap water for 1 min before differentiation with 0.7% hydrochloric acid in ethanol for about 5 s. The section was rinsed with water for 5 min. Eosin staining was performed for 1 min, and rinsed with tap water for 2 min. The sections were dehydrated though a graded series of alcohol concentrations at 75%, 85%, 95%, dehydrated alcohol, and dehydrated alcohol II (each for 3 min), and superfluous fluid was absorbed with absorbent paper between the different dehydration steps. The sections were vitrified with xylene I for 5 min and xylene II for 5 min. They were mounted under glass coverslips with neutral gum, and observed using an optical microscope.

### Immunohistochemistry

Tissue sections were attached to glass slides with poly-L-lysine and baked at 65 °C for 4.5 h. Then the section was soaked in xylene for 30 min, rinsed with 100% alcohol twice, with 95% alcohol once, and with 80% alcohol once, followed by tap water. We added 4.3% methanol perhydrol at room temperature for 20 min, rinsed with distilled water, and then rinsed with 0.1 M phosphate-buffered saline (PBS). Antigen retrieval was performed by incubating the sections with antigen retrieval buffer (EDTA buffer pH 9.0; catalog No. ZLI-9086, Zsgb-Bio) in a beaker placed in a waterbath at a sub-boiling temperature for 20 min, before cooling for 3–5 min with tap water. Wax was painted on the slide to confine droplets of goat serum applied and incubated with the section at room temperature for 20 min to block nonspecific binding sites. Unnecessary liquid was discarded without rinsing. Primary antibody against connective tissue growth factor (CTGF) (polyclonal rabbit anti-CTGF antibody, product No. bs-0743R; Beijing Bioss Biotechnology Co.; RRID: AB_10856885) or (anti-TGF β1 monoclonal antibody from mouse, clone number 12E5, 1:100, product No. bsm-33287M; Beijing Bioss Bio-technology Co.) was added at 4 °C overnight, rinsed with 0.1 M PBS for 5 min/3 times. Secondary antibody (biotin-labeled goat anti-rabbit IgG polymer, product Nos. SP-9001, SP9002, SP kit, rabbit streptavidin–biotin method detection system, Beijing Zsgb-Bio; RRID: AB_2758396) was added at 37 °C for 20 min, rinsed with 0.1 M PBS for 5 min/3 times. Cochlearia enzyme (horseradish peroxidase (HRP)) labeled strepto-albumin fluid (S-A/HRP, SP kit SP9001, rabbit streptavidin-biotin method detection system, Beijing Zsgb-Bio) was added at 37 °C for 20 min, rinsed with 0.1 M PBS for 5 min/3 times. A diaminobenzidine (DAB) chromogen kit (HeBei Bohai Bioengineering Exploitation Co.) was used to develop staining and the section was rinsed with water. Hematoxylin counterstaining of the nucleus was performed for 1 min and rinsed with water sufficiently. The staining was differentiated by removing excess dye with 1% HCl in ethanol and rinsing the section with water sufficiently. Then 1% ammonia water was added to differentiate the staining by bluing the color of the hematoxylin-stained nuclei, then the sections were rinsed with water sufficiently, and dehydrated through a graded series of alcohols 80%, 95%, and 100% alcohol twice. The sections were vitrified with xylene for at least for 30 min, and mounted under glass coverslips with neutral resin. The image was analyzed with a CellSens Standard pathology image analysis system (version 1.12; Olympus, Japan). The section was observed with a light microscope (×200). The venous wall was observed and the thickness of the tunica intima (intimal thickness was the distance from endothelium to internal elastic lamina) was recorded. We measured at 4 places from every section to obtain a mean value. The corresponding immunohistochemistry score was calculated. Standardized score: 10 nonrepetitive visual fields under ×200 were selected without preference in each slice, and percentage of positive cell and staining degree in each visual field were determined. The scores for positive cell percentage were: 0: stained area <1%; 1: stained area 1%–10%; 2: stained area 10%–50%; 3: stained area 50%–80%; 4: stained area >80%, diffuse staining. The degree of staining scores were: 0: no staining; 1: light yellow; 2: yellowish-brown; 3: brown [15].

### Statistical analysis

Data were analyzed using SPSS for Windows (version 16.0; SPSS Inc). A repeated measures analysis of covariance (ANCOVA) was used for adjusted baseline (weight, dose of pentobarbital, operating time, preoperative diameter). *P* < 0.05 was considered significant.

## Results

### Comparison of rabbit data between the 2 groups

The mean weight of the rabbits between the 2 groups was not significantly different. The dose of 1% sodium pentobarbital in hydraulic expansion group was not significantly different from that used in the control group. The operating time in hydraulic expansion group was significantly longer than in the control group (*P* = 0.013). The preoperative arterial and venous diameters measured by B-mode ultrasonic diagnostic equipment were not significantly different between rabbits in the hydraulic expansion and control groups (**Table 1**).

**Table 1:** Baseline data for rabbits in experimental and control groups.

	**Hydraulic expansion (n = 6)**	**Control (n = 6)**
Weight (kg)	2.2 ± 0.1	2.26 ± 0.11
Dose 1% pentobarbital (mL)	8.67 ± 1.44	7.38 ± 0.88
Operating time (min)	150 ± 20^*^	84 ± 20.8
Artery diameter, preoperative (mm)	1.73 ± 0.18	1.8 ± 0.21
Vein diameter, preoperative (mm)	2.73 ± 0.51	2.48 ± 0.65

* *P* < 0.05 compared with control group (Student *t* test).

The diameter of the fistula vein was increased significantly after surgery. After adjusting for weight, dose of pentobarbital, operating time, and preoperation diameter, the diameter of the fistula vein in hydraulic expansion group immediately after surgery (baseline) was significantly greater than it was in the control group (*F*_4,11_ = 6.79, *P* = 0.02). At 1 day after surgery, the diameter of the fistula vein in hydraulic expansion group was also significantly greater than it was in those rabbits of the control group (*F*_4,9_ = 7.87, *P* = 0.03). However, the diameter of fistula vein between the groups at 2, 3, 7, 14, or 21 days after surgery was not significantly different (**Table 2**). Vasospasm occurred in one rabbit in the hydraulic expansion group, and reduced after spasmolysis with topical lidocaine. One rabbit had an infection at the incision site that healed after changing dressings. Death as a result of anesthesia overdose occurred in another rabbit that was replaced with another to maintain group size.

**Table 2:** Diameter of fistula vein after AVF surgery in rabbits

**Postoperative day**	**Hydraulic expansion (n = 6)** **Diameter (mm)**	**Control (n = 6)** **Diameter (mm)**	***F***	***P***
Baseline (0)	4.34 ± 0.50	2.80 ± 0.57	6.79	0.02^*^
1	4.02 ± 0.58	3.01 ± 0.18	7.87	0.03^*^
2	3.92 ± 0.91	3.34 ± 0.26	0.97	0.53
3	5.12 ± 1.30	4.36 ± 0.86	1.72	0.28
7	4.93 ± 0.52	4.17 ± 0.51	1.94	0.22
14	5.19 ± 1.04	4.18 ± 0.52	0.99	0.51
21	4.52 ± 0.29	4.39 ± 0.28	0.95	0.52

ANCOVA, analysis of covariance; AVF, arteriovenous fistula.

* *P* < 0.05, repeated measures ANCOVA, adjusting for weight, anesthesia dose, operation time, and preoperative vessel diameter.

### Histomorphology

After hydraulic expansion and before construction of the fistula, the wall of the vein was thinner in macroscopic observation and the anterior and posterior wall were difficult to distinguish during surgery in the hydraulic expansion group. Saline seepage was caused by extreme dilation, but blood did not leak. At 3 weeks after surgery, the incisions in rabbits of both groups healed well. The blood vessel of the fistula could be felt, and the wall of vessel was thickened and there was slight adhesion to surrounding tissue, which could be separated easily.

### Morphology of tissue section by hematoxylin and eosin staining

**Figure 2:**
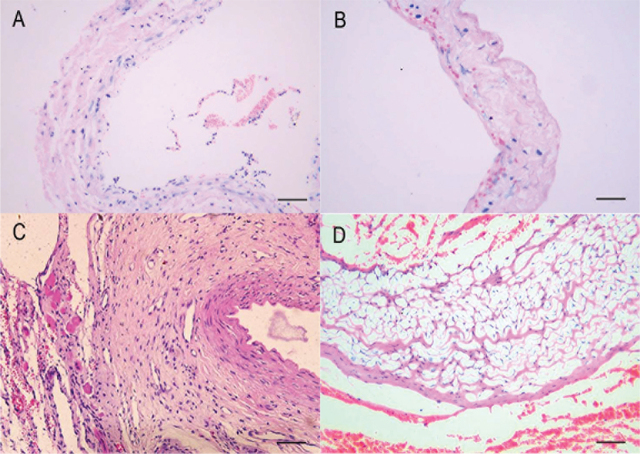
Histology. **(A)** Venous wall before dilation. **(B)** Venous wall after dilation. **(C)** Changes of fistula vein in control group 3 weeks after operation. **(D)** changes of fistula vein in hydraulic expansion group 3 weeks after operation (all panels HE staining, ×200; scale bars represent 50 μm). HE, hematoxylin and eosin.

The changes of the venous wall are shown before and after dilation (**Figure 2A** and **B**). Before the hydraulic expansion, the venous endothelium had integrity without dilation. The internal elastic lamina was clear. There were a few circular smooth muscle cells in the tunica media. The tissue was comparatively compact. There were longitudinal smooth muscle cells in the tunica externa. The external elastic lamina was not clear and boundary was obscure (**Figure 2A**). After hydraulic expansion, the structure of venous wall was deranged, the endothelium was disrupted and then sloughed off, circular smooth muscle cells under the epithelium were exposed, elastic fibers were disrupted and deformed. A large number of erythrocytes had entered the tunica media and tunica externa, the wall was compact and thinner, and the boundary of tunica externa was clear (**Figure 2B**).

At 3 weeks after surgery, the venous wall of fistula had thickened in control group, intimal hyperplasia was obvious, endothelial cells protruded into the lumina, smooth muscle cells had proliferated, cell number was increased in each layer, and the density of tissue was greater than it was before surgery (**Figure 2C**). In the hydraulic expansion group, the venous wall of fistula was thickened, intimal hyperplasia was obvious, most of cells were smooth muscle cells, and cells were arranged chaotically. There were fewer elastic fibers than seen in the control group. The smooth muscle cells in the tunica externa had proliferated. The tissues from the endothelium to the tunica externa in hydraulic expansion group were rarefied compared with tissues in the control group. There was less extracellular matrix in the hydraulic expansion group than in the control group (**Figure 2D**).

### Expression and immunohistochemistry scores of transforming growth factor-β1 (TGF-β1) and CTGF

**Figure 3:**
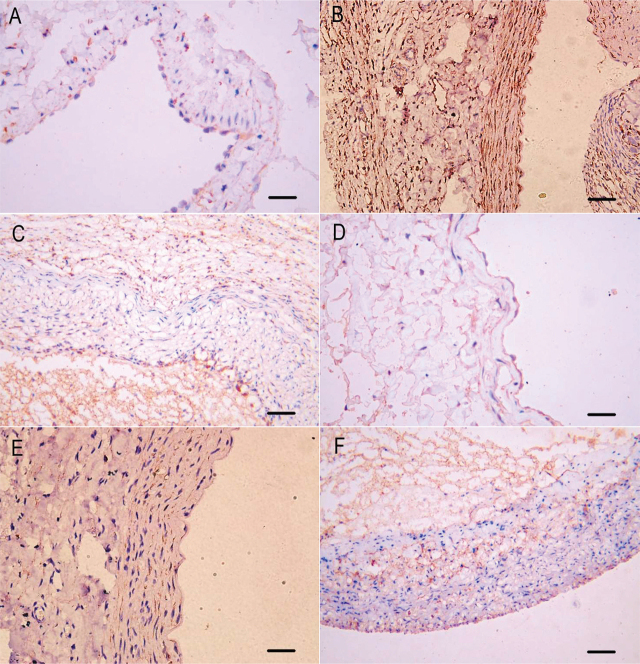
Expression of TGF-β1 immunoreactivity in vein (A) before surgery, (B) fistula vein in control group 3 weeks after surgery, **(C)** fistula vein in hydraulic expansion group 3 weeks after surgery. Expression of CTGF immunoreactivity in vein **(D)** before surgery, **(E)** fistula vein in control group 3 weeks after surgery, **(F)** fistula vein in hydraulic expansion group 3 weeks after surgery. (DAB staining of immunocomplexes, ×200; scale bars represent 50 μm). CTGF, connective tissue growth factor; DAB, diaminobenzidine; TGF-β1, transforming growth factor-β1.

The expression of transforming growth factor-β1 (TGF-β1) and CTGF (polyclonal rabbit anti-CTGF antibody, product No. bs-0743R; Beijing Bioss Biotechnology Co.; RRID: AB_10856885) were mainly located in cellular cytoplasm (**Figure 3**). In the vein before surgery, TGF-β1 immunoreactivity was expressed in endothelial cells, but there was only weak expression (yellowish-brown staining) in the tunica media and tunica externa (**Figure 3A**). Three weeks after surgery TGF-β1 immunoreactivity was expressed in the tunica intima, tunica media, and tunica externa, and appeared as yellowish-brown staining in the control group (**Figure 3B**). TGF-β1 immunoreactivity was expressed in the tunica intima and tunica externa, but only slightly in the tunica media, and appeared as yellowish-brown staining in the hydraulic expansion group (**Figure 3C**). CTGF immunoreactivity was expressed clearly in endothelial cells, but was weak in the tunica media and tunica externa appearing as yellowish-brown staining (**Figure 3D**). Three weeks after surgery, CTGF was expressed clearly in tunica intima and tunica externa, but was stronger in the tunica intima, and appeared as yellowish-brown staining in the control group (**Figure 3E**). CTGF immunoreactivity was expressed in tunica externa, but weakly in the tunica intima and tunica media, and appeared as yellowish-brown staining in the hydraulic expansion group (**Figure 3F**). After adjusting for the baseline (weight, dose of pentobarbital, operating time, pre-operative diameter), there was no difference in intimal thickness between 2 groups 3 weeks after surgery (*F*_4,11_ = 0.95, *P* = 0.51). Immunohistochemistry scores for TGF-β1 in hydraulic expansion group were less than those in the control group, but the difference was not significant (*F*_4,11_ = 0.60, *P* = 0.71). Immunohistochemistry scores for CTGF in the hydraulic expansion group were significantly less than those in the control group (*F*_4,11_ = 7.87, *P* = 0.01) (**Table 3**).

**Table 3:** Intimal thickness and immunohistochemistry score 3 weeks after surgery

	**Hydraulic expansion (n = 6)**	**Control (n = 6)**	***F*_4,11_**	***P***
Intimal thickness (mm)	39.20 ± 3.78	39.34 ± 1.91	0.95	0.51
Expression of TGF-β1 (score)	4.32 ± 0.31	5.32 ± 0.91	0.60	0.71
Expression of CTGF (score)	3.70 ± 0.05	4.90 ± 0.09	7.87	0.01^*^

CTGF, connective tissue growth factor; TGF-β1, transforming growth factor-β1.

## Discussion

After the construction of an AVF, a series of pathophysiological changes are induced in the autogenous vein under the high artery flow and pressure. The vein shows outward expansion and inward hyperplasia. Outward expansion is an adaptation reaction, also known as venous arterialization. The venous wall thickens and the venous lumen is expanded. This allows the fistula to mature gradually and achieves the requirement for cannulation for hemodialysis. Inward hyperplasia is an over response. Intimal hyperplasia can lead to fistula stenosis, lumens to be thickened, and fistula function to be lost [16, 17]. In our present study, visual observation found that the venous vessels were thickened and the lumens were expanded after the construction of an AVF in rabbit. Ultrasonography showed expanded lumens and multicolor signal from turbulent blood flow at the anastomotic stoma. This is similar to the changes of human vein observed after construction of an autogenous artery–venous fistula. In the present study, there was a single surgeon and the healthy rabbits had normal renal function, so the effect of surgical variance or uremia on the vein was excluded. It may be useful to observe the effect on venous post injury of fistula vascular endothelium and smooth muscle cell after a different surgical method.

The operation time in the hydraulic expansion group was longer than it was in the control group. The increase in time is due to the increased difficulty in surgery after the vein was dilated and became thinner. The venous diameter in the hydraulic expansion group was significantly greater compared with the diameter in the control group at 1 day after construction of the fistula. This suggests that the enlarged venous diameter was maintained for a short while after hydraulic expansion surgery and this was useful to prevent early thrombosis at the fistula. A study of 17 patients found that the greater venous distensibility was associated with better AVF maturation [18]. HE staining showed the tunica media and adventitia were disrupted in the dilated vein. This may be related the state of enlargement. A mouse model showed that elastin has an important role in vascular remodeling after an AVF was created, in which a lower amount of elastin resulted in enhanced outward remodeling [19]. In the present study, there were fewer elastic fibers in the fistula vessel in the hydraulic expansion group 21 days after surgery, and this was helpful for outward extension of the fistula and to maintain its patency.

The vascular trauma from venipuncture and intravenous peripheral lines may induce pre-existing vascular pathologies, but it cannot explain the extensive intimal hyperplasia frequently found in distal saphenous veins that had never been punctured or traumatized [20]. This indicates that trauma is not the single factor for intimal hyperplasia. In the present study, the degree of intimal hyperplasia in the hydraulic expansion group was similar to that in the control group. This indicates that the effect of trauma from hydraulic expansion on the vein was not greater than the effect of hemodynamic changes on the vein. Thus, hydraulic expansion alone did not increase intimal hyperplasia.

AVF access stenosis has been characterized histologically as having a role in intimal hyperplasia [16, 17]; vascular smooth muscle cells (VSMC) induced intimal expansion via a rapid synthesis of extracellular matrix; its proliferation and migration were mediated by several growth factors, including TGF-β1 and CTGF [21]. As a common multifunctional cytokine, TGF-β1 assumes a role in regulating the proliferation and differentiation of various cells [22]. The effect of TGF-β1 is to bind to cell surface receptors, and 3 receptors including VSMC have been found in most cells. TGF-β1 is important for the start and end of wound healing after tissue injury [23]. Increased or decreased TGF-β1 is associated with numerous pathologies [24]. TGF-β1 can inhibit migration and proliferation of macrophages and protect endothelial function [25], is a recognized promoter in VSMC proliferation and induces fibrosis and ECM production in blood vessels [26, 27]. By inducing the synthesis of extracellular matrix proteins, overexpression of TGF-β1 may promote the development of intimal hyperplasia and induce fistula stenosis and subsequent access failure due to synthesis of extracellular matrix proteins [28]. CTGF is downstream factor of TGF-β1, and plays a role in promoting the fibrosis induced by TGF-β1. CTGF can promote cell mitosis, induce cell mitosis and proliferation, induce adhesion, and promote ECM production [29]. Thus, detecting expression of TGF-β1 and CTGF in fistula venous tissue can reflect any change of intimal proliferation. In the present study, TGF-β1 and CTGF were clearly expressed in the fistula vasculature in the control group, but their expression was weaker in the hydraulic expansion group. This weaker expression was associated with a decrease of extracellular matrix in the hydraulic expansion group suggesting that changes of hemodynamics stimulated intact veins, caused TGF-β1 and CTGF expression and intimal proliferation; but to disorganized venous wall structure in the hydraulic expansion group, TGF-β1 and CTGF expression was weaker and was concentrated in the tunica externa. Classically smooth muscle cells transfer from the tunica media and tunica externa to the tunica interna. The present study suggested that hydraulic expansion can influence the process of intimal proliferation by reducing the expression of TGF-β1 and CTGF, but does not increase intimal hyperplasia of the fistula vasculature. Hydraulic expansion does not apparently increase the risk of nonthrombogenic stenosis of an AVF.

This study in rabbits is limited by its small sample size. Further studies with larger sample are necessary to verify present findings. The responses in the rabbit model of AVF may not be applicable to humans who may have different endothelial responses due to species differences. The observation time was relatively short and may not accurately reflect long-term vascular intimal changes. The present experiments studied the effects of hydraulic expansion on blood vessels in healthy rabbits without uremia. Further studies that accommodate the possible effects of uremic toxins are warranted.

## Conclusions

Hydraulic expansion does not increase the intimal hyperplasia of an AVF and is helpful for outward remodeling of the AVF. This technique does not increase the risk of nonthrombogenic stenosis of the AVF and may increase its primary patency. In the present study, visual observation found that the venous blood vessels were significantly thickened after surgery, and the lumen was significantly dilatated. Ultrasonography also indicated that the vascular lumen is enlarged and turbulent blood flow signals were seen at the anastomosis. The anastomosis of cervical vessels in rabbit may simulate human autogenous AVF.
